# Current Radiotherapy Considerations for Nasopharyngeal Carcinoma †

**DOI:** 10.3390/cancers14235773

**Published:** 2022-11-24

**Authors:** Wai Tong Ng, James C. H. Chow, Jonathan J. Beitler, June Corry, William Mendenhall, Anne W. M. Lee, K Thomas Robbins, Sandra Nuyts, Nabil F. Saba, Robert Smee, William A. Stokes, Primož Strojan, Alfio Ferlito

**Affiliations:** 1 Clinical Oncology Center and Shenzhen Key Laboratory for Cancer Metastasis and Personalized Therapy, The University of Hong Kong-Shenzhen Hospital, Shenzhen 518009, China; 2 Department of Clinical Oncology, Li Ka Shing Faculty of Medicine, The University of Hong Kong, Hong Kong SAR, China; 3 Department of Clinical Oncology, Queen Elizabeth Hospital, Hong Kong SAR, China; 4 Knox County Health Clinic, Rockland, ME 04841, USA; 5 Division of Radiation Oncology, GenesisCare Radiation Oncology, St. Vincent’s Hospital, Melbourne, VIC 3065, Australia; 6 Department of Radiation Oncology, University of Florida College of Medicine, Gainesville, FL 32610, USA; 7 Department of Otolaryngology-Head and Neck Surgery, Southern Illinois University School of Medicine, Springfield, IL 62794-9620, USA; 8 Department of Radiation Oncology, University Hospitals Leuven, KU Leuven—University of Leuven, 3000 Leuven, Belgium; 9 Department of Hematology and Medical Oncology, Winship Cancer Institute, Emory University School of Medicine, Atlanta, GA 30322, USA; 10 Department of Radiation Oncology, The Prince of Wales Cancer Centre, Sydney, NSW 2031, Australia; 11 Department of Radiation Oncology, Winship Cancer Institute, Emory University School of Medicine, Atlanta, GA 30322, USA; 12 Department of Radiation Oncology, Institute of Oncology, 1000 Ljubljana, Slovenia; 13 Coordinator of the International Head and Neck Scientific Group, 35100 Padua, Italy

**Keywords:** nasopharyngeal cancer, radiotherapy, target delineation, adaptive planning, artificial intelligence, particle therapy

## Abstract

**Simple Summary:**

Nasopharyngeal carcinoma (NPC) is commonly treated using high-dose radiotherapy. Careful radiotherapy planning is crucial for the eradication of cancer cells while avoiding injuries to normal structures. This balance is often delicate given the complex anatomic location in which NPC is situated. This article highlights the considerations, practical pearls, and recent advances in the precise delivery of radiotherapy in NPC patients.

**Abstract:**

Radiotherapy is the primary treatment modality for nasopharyngeal carcinoma (NPC). Successful curative treatment requires optimal radiotherapy planning and precise beam delivery that maximizes locoregional control while minimizing treatment-related side effects. In this article, we highlight considerations in target delineation, radiation dose, and the adoption of technological advances with the aim of optimizing the benefits of radiotherapy in NPC patients.

## 1. Introduction

Radiotherapy is the key treatment modality for nasopharyngeal carcinoma (NPC). With the advent of intensity-modulated radiotherapy (IMRT) and refinements to different imaging modalities, the precision of radiation delivery has significantly improved in the recent decade, giving rise to favorable tumor control and toxicity outcomes in contemporary cohorts [1,2]. International consensus guidelines have emerged to harmonize variations in tumor target delineation and to guide dose prioritization for NPC radiotherapy [3,4]. Since then, researchers have been active in further optimizing the therapeutic ratio by refining the delineation of tumor targets and organs at risk (OAR) and personalizing radiation prescription doses. This article aims to provide a comprehensive update on the recent advances in definitive radiotherapy for NPC and highlights potential future developments in this field.

## 2. Target Delineation

Target delineation in radiotherapy for NPC is highly challenging given the complex local anatomy, tumor spreading pattern, and the intricate relationship between tumor targets and multiple critical OAR. An accurate, cautious, and rational target delineation process is crucial to attaining satisfactory loco-regional control and long-term cure. Special efforts are also required in the proactive sparing of normal structures to minimize the incidence and severity of radiation-associated complications, many of which may pose lifelong detriments to the quality of life of survivors.

With the advent of high-resolution fiberoptic endoscopy, magnetic resonance imaging (MRI), and positron emission tomography (PET), the accuracy of the gross tumor volume (GTV) delineation for both primary nasopharyngeal tumors and regional lymph nodes has greatly improved. Input from diagnostic radiologists specializing in head and neck cancers is of vital importance in ensuring the accurate interpretation of all pre-treatment staging images. The challenge for radiation oncologists now resides in determining the optimal extents of high- and low-risk clinical target volumes (CTV), which could vary significantly depending on the presenting stage of disease as well as institutional practices and consensus guidelines [4,5]. Regular quality assurance programs and peer reviews also play an essential role in ensuring correct target delineation and satisfactory dosimetry [6].

Traditionally, CTV delineation for NPC has relied on historical studies which reported the natural route of spread with the aim of prophylactically treating areas that were included within the radiation portals used in the era of two-dimensional radiotherapy. In 2018, a set of international consensus guidelines were published to instruct CTV delineation for NPC [4]. They defined high-risk CTV (CTV1) as a 5 mm volumetric expansion from GTV, whereas low-risk CTV (CTV2) includes a further 5 mm expansion from CTV1. These CTVs were then manually edited to cover specific anatomical regions according to the eccentric local spread pattern with the exclusion of natural barriers. For gross tumors encroaching onto critical neurological structures at risk, a tighter CTV margin of 1–2 mm was recommended as a necessary trade-off to balance tumor control and the risk of complications. While this work represents the first international collaborative effort to harmonize highly heterogeneous target delineation practices for NPC, there are a few limitations to note. First, the current “5 + 5” recommendation of GTV expansion was largely extrapolated from pathologic evidence of microscopic spread observed in other, non-nasopharyngeal head and neck squamous cell carcinomas, which exhibit distinct biological behavior from NPC [7]. Second, the expert consensus level in this guideline was relatively low in several areas. For example, agreement for CTV1 to cover the whole nasopharynx was only 55%, and only 3/4 of the voters agreed on the additional 5 mm volumetric expansion from CTV1 to CTV2 [4]. This highlighted the divergent preferences in CTV delineation among international experts. Third, this guideline was meant to standardize delineation practice, and clinical data on disease control and toxicities using this approach were unavailable at that time.

It is noted that while CTV delineation based on “5 + 5” expansion margins and anatomic landmarks represents a one-size-fits-all approach that prioritizes tumor coverage, the final treatment volumes tend to be larger, potentially leading to a higher risk of acute and late complications. Given the growing understanding of failure patterns and the increasing awareness of survivorship issues in NPC patients, there have been ongoing efforts to individualize CTV delineation according to various tumor extents.

### 2.1. CTV Delineation of Primary Tumors

The intricate anatomy immediately surrounding the nasopharynx requires the precise delineation of gross primary tumors and corresponding target volumes. A multimodal imaging approach including contrast-enhanced computer tomography, gadolinium-enhanced magnetic resonance imaging (MRI), and ^18^F-fluorodeoxyglucose positron emission tomography (PET) offers the most comprehensive anatomic depiction of tumor extent. These diagnostic studies can be imported into radiation treatment planning systems and registered with simulation scans to facilitate contouring target volumes.

The international CTV delineation guidelines recommended the symmetrical inclusion of structures at risk of microscopic spread regardless of the laterality of the primary tumor [4]. One area of active research is the selected sparing of the contralateral structures in patients presenting with unilateral disease. As the fossa of Rosenmüller is the most common site of origin, early NPC typically presents unilaterally and may remain so as the tumor advances, constituting approximately 10% of all NPC at presentation [8,9]. An endoscopic biopsy study has revealed a low incidence of occult microscopic disease in the contralateral nasopharyngeal mucosa in unilateral NPC which did not cross the midline [8]. The risk of skipped discontinuous invasion to the contralateral parapharyngeal space and skull base is also exceedingly low in unilateral NPC, making unilateral irradiation an attractive option to reduce radiation side effects, such as trismus and xerostomia [10].

In a retrospective report of 95 unilateral NPC, unilateral irradiation was delivered by delineating CTV2 as a 15–20 mm volumetric expansion from GTV with deliberate exclusion of contralateral nasopharyngeal mucosa and parapharyngeal space and skull base structures [11]. This contouring approach has led to an excellent 10-year local recurrence-free survival rate of 96.2%, in which no out-field recurrence occurred contralaterally. Importantly, mean radiation doses to the contralateral organs at risk, such as the parotid, middle ear, and temporomandibular joint, were 13–33% lower than those of their ipsilateral counterparts, and no significant late toxicity was reported. In another study, Sanford et al. reported the clinical outcomes of 73 patients who were treated using a CTV delineation method individualized to the primary tumor extent [12]. Apart from limiting CTV2 to the ipsilateral parapharyngeal space, pterygopalatine fossa, and foramen ovale in lateralized tumors, there was no routine inclusion of the clivus, nasal cavity, maxillary sinus, ethmoid sinus, or sphenoid sinus unless these structures were involved. Compared to the standard contouring protocol in the NRG HN001 trial, this target delineation approach resulted in a 36–62% reduction in CTV2 volume and radiation doses to multiple normal structures. The 5-year local control rate was high at 94% and all loco-regional recurrences occurred within the high-dose CTV.

Another area of active research is the reduction in CTV upon response to induction chemotherapy. In recent years, there has been increasing evidence to support the adoption of extended chemotherapy in advanced NPC, demonstrating significant improvement in both recurrence-free survival and overall survival compared to chemo–radiotherapy alone [13,14]. Traditionally, with the concern that tumor regression after chemotherapy is not uniformly eccentric, the conventional practice was to treat all pre-chemotherapy gross tumors to a full therapeutic radiation dose of at least 70 Gy [4]. However, this approach often results in large treatment volumes with potentially unnecessary toxicities. Retrospective evidence in T4 NPC supports the feasibility and safety of restricting the 70 Gy volume to the post-chemotherapy disease extent while treating the pre-chemotherapy extent with 60 Gy, after which no out-field local failure was reported [15]. A similar approach has been reported in a phase II de-escalation trial, with part of the post-chemotherapy GTV receiving a moderate dose reduction of 66 Gy for T3–4 diseases [16]. This adaptive CTV delineation approach was also tested in a phase III randomized controlled trial [17]. In the experimental arm of this trial, CTV1 (64 Gy; GTV boosted to 70 Gy) covered a 0.5–1 cm margin from the post-chemotherapy tumor, and CTV2 (54 Gy) covered all at-risk regions plus the pre-chemotherapy tumor extent. Compared with conventional contouring, this approach markedly reduced the mean volume of CTV1 from 366 cc to 305 cc. Patients who were treated with adaptive target delineation had a 2-year overall survival comparable to controls, but with a lower incidence of dry mouth and better quality of life metrics. The results of a recent prospective phase II study also confirmed long-term efficacy with this approach and reported no out-field local recurrence at a median follow-up of 10 years [18]. It is noted that all of the above studies treated skull base disease according to the pre-chemotherapy extent as the tumor response in bony structures is challenging to ascertain.

### 2.2. CTV Delineation of Regional Lymphatics

NPC has a high propensity for nodal metastasis, with more than 50% of patients presenting with advanced nodal disease [1]. In a meta-analysis of patients totaling 2920 NPC cases who were staged using MRI, 85% were found to have lymphadenopathy, among whom 69% had retropharyngeal nodes and 70% had nodal disease in level II [19]. In this context, the close proximity of the retropharyngeal nodal basin to the nasopharynx deserves special consideration. These lymph nodes lie within a fat pad located behind the posterior wall of the oropharynx and nasopharynx. Behind them lies the prevertebral fascia. The nodes can extend caudal from the skull base to the level of the carotid bifurcation. The medial retropharyngeal nodes lie in the midline at a level between the first and fourth cervical vertebrae, whereas the lateral nodes are located immediately medial to the internal carotid artery. The nodes are supplied by afferent lymphatics from the nasal cavity, nasopharynx, and eustachian tubes [20]. Their primary efferent channels extend towards the deep cervical nodes that lie along the internal jugular vein in level II. Lin et al. evaluated 1000 NPC patients for nodal disease involving selected levels [21]. In a total of 10,651 nodes imaged, there were 819 nodes detected in the newly classified level VIIA, and 5 of them involved the medial retropharyngeal nodes.

Prior to imaging techniques that adequately depicted the medial and lateral retropharyngeal lymph nodes, it was common understanding that lymph nodes in level II were the most common sites for clinical metastases from NPC. Although the French anatomist Henri Rouvière documented the retropharyngeal lymphatics through anatomical dissections, there remained insufficient evidence to depict retropharyngeal nodal metastases clinically unless they were markedly enlarged. Since the advent of CT, MRI, and FDG-PET scans, retropharyngeal node involvement with NPC is more commonly appreciated. Consequently, the retropharyngeal lymphatics with their nodal basin, both medial and lateral, have become recognized as the primary echelon for lymphatic drainage from the nasopharynx. However, level II may also be the first echelon lymphatic basin without retropharyngeal node involvement, especially for primary diseases that extend laterally.

The lymphatic spread of NPC typically follows a stepwise pattern, starting from the upper basins, including the retropharyngeal and level II nodes, and then extending caudally along the deep cervical (jugular) and spinal accessory nodes. Ho FC et al. showed that the percentage of patients with positive nodal involvement in levels III, IV, and V was determined to be 45%, 11%, and 27%, respectively. Among low-risk nodal basins were the supraclavicular nodes, level I, level VI, and the parotid gland, all of which varied from 0% to 3%. The authors reported that the probability of skip metastasis between levels varied between 0.5 and 7.9%, leading to the suggestion that a reduced treatment volume for the elective irradiation of subclinical NPC nodal disease is feasible [19].

Toward a more refined approach for reduced treatment volume based on the risks of occult nodal disease, CTV delineations have become based on the international consensus agreement in 2013 intended to minimize inter-observer variation in nodal delineation for head and neck cancers [22]. Since then, researchers have tried to further refine the delineation boundaries according to the unique nodal spread pattern of NPC. In one study which investigated the distribution of 10,665 involved cervical nodes of patients with NPC, it was shown that the consensus atlas missed 13% of the nodes posteromedial to the level Vb boundaries and 1.5% of the nodes cranial to the level VIIa boundaries [22]. By contrast, no cervical node involvement was identified in the following specific areas: within the submandibular gland; the gap between the sternocleidomastoid and splenius muscles in level II; the gap between the sternocleidomastoid and infrahyoid muscles in level IVa; and the gap between the skin and omohyoid muscle in level Vc. Therefore, the NPC-specific delineation of the cervical lymphatics was proposed to improve coverage and reduce unnecessary normal tissue irradiation, which contributes to neck fibrosis and post-radiation hypothyroidism [23].

The conventional delineation of level Ib includes the entire submandibular gland and a larger area of soft tissue between the oral cavity and the mandible, the irradiation of which leads to substantial salivary dysfunction and long-term oral complications. The current guidelines recommend limiting the prophylactic irradiation of level Ib nodal basins only when tumors invade the submandibular gland, oral cavity or nasal cavity, and in situations where level IIa nodes are large or demonstrate features of extracapsular extension [4]. Modifications of the level Ib nodal CTV boundaries have been specifically proposed for NPC. In one nodal topographic study of 54 NPC patients with positive level Ib nodes, researchers reported a very low risk of nodal occurrence within and medial to the submandibular glands, whereas most radiologically abnormal nodes were found eccentrically above or below the glands [24]. By limiting the elective level Ib boundaries to the soft tissues within specific distances (11 mm superiorly and 17 mm inferiorly) but not intentionally targeting the submandibular glands, radiation volumes could be significantly reduced compared to those when using the standard consensus atlas, which may attenuate the excess dose to the salivary glands, mandible, and the oral cavity mucosa.

International consensus guidelines have recommended the prophylactic irradiation of level Ib nodal basins when level II nodes are sizable (>2 cm) or exhibit radiological evidence of extracapsular extension. Recently, a large propensity score–matched analysis evaluated the feasibility of sparing level Ib nodal basins in these traditionally high-risk scenarios. It was shown that the rate of level Ib failure remained exceptionally low (1.8%), and its omission could lead to a lower incidence of xerostomia [25]. These new data support a more stringent selection of patients for prophylactic level Ib irradiation, prompting a potential revision of the current target delineation guidelines.

Apart from the efforts in refining the definitions of nodal levels, several studies have been reported on limited neck irradiation in selected cases of NPC. The omission of lower neck irradiation in the uninvolved neck was proved safe in NPC in a recent phase III randomized non-inferiority trial [26]. This study randomized 446 patients with N0 or N1 (retropharyngeal node only) NPC into prophylactic whole neck irradiation (level II, III, IVab and Vab) or upper neck irradiation alone (level II, III and Va). The 3-year regional relapse-free survival rates were comparable between the two arms (96.3% vs. 97.7%). Importantly, sparing the uninvolved lower necks led to lower incidences of late toxicities, including hypothyroidism, skin complications, dysphagia, and neck tissue damage. This is important evidence supporting the routine omission of lower neck irradiation in low-risk NPC.

## 3. Optimizing Prescription Doses

### 3.1. Dose De-Escalation

Concerning the prescription dose of definitive radiotherapy for NPC, most head and neck oncologists practice in accordance with international consensus guidelines by giving a dose of 70 Gy to high-risk CTV and 50–60 Gy to low- to intermediate-risk CTV [4]. Treatment is typically delivered using the simultaneous integrated boost technique with different radiation doses delivered to target volumes within a single treatment fraction. With contemporary imaging modalities, such as MRI and PET, the definition of tumor extent and sensitivity in detecting pathologic nodes have significantly improved, leading to the proposal of a “gradient-dose” concept in dose prescription for head and neck squamous cell carcinomas [27,28]. Instead of the conventional binary two-tier CTV system, this concept advocates the focus of high-dose radiotherapy only on gross tumors identified by contemporary imaging along with the delivery of a decreasing radiation dose over a defined distance from the main disease, thereby reducing radiation toxicity.

Similar radiation dose prescription approaches have long been practiced by multiple oncology centers for NPC in China. The full therapeutic radiation dose of 70 Gy was limited to the GTV with no additional CTV margin, followed by 60 Gy and 50–54 Gy for intermediate-risk and low-risk CTV, respectively [5]. In one prospective observational study of 471 patients, by restricting the radiation dose of 66–70 Gy to GTV and 54–56 Gy to CTV (8 mm isocentric expansion from GTV) with an additional 3 mm planning target volume margin on each volume, an excellent 4-year local recurrence-free survival rate of 96.6% was reported with no in-field failure [29]. Compared to the prescription recommendations in the international guidelines, this de-escalated prescription approach restricts the zone of high-dose radiation, potentially reducing radiotherapy complications in NPC patients while apparently maintaining oncologic safety.

The next key question is whether a high radiation dose of 70 Gy is necessary to attain a cure for all NPC. A retrospective propensity score–matched analysis on a small series of 32 T1–T3 NPC patients with incomplete radiotherapy at doses of 53–67.8 Gy reported comparable 5-year loco-regional relapse-free survival with patients who received the full 70 Gy (92.5% vs. 91.7%) [30]. Albeit retrospective, this evidence suggested that the uniform conventional dose prescription may not be necessary for radiosensitive tumors such as NPC. In the same vein, a logical approach by which to attempt dose de-escalation is to focus on tumors that demonstrate favorable responses to induction chemotherapy. Clinical trials of pediatric NPC have reported a satisfactory 5-year event-free survival rate of 77–91% with 45–68 Gy of radiotherapy following induction chemotherapy, and the dose could be safely reduced from 59.4 Gy to 54.4 Gy for patients who attained complete remission on MRI and PET [31,32]. Comparable survival outcomes between dose-reduced (60–65.9 Gy) and standard-dose (66–72 Gy) IMRT following favorable responses to induction chemotherapy have also been reported in a recent pediatric NPC study [33]. Furthermore, in a phase II clinical trial from China, 216 adult patients with stage III NPC who had low pre-treatment Epstein–Barr virus (EBV) DNA levels (<4000 copies/mL) were treated with 60 Gy of radiotherapy after a complete or partial response to induction chemotherapy [34]. This approach yielded an encouraging 2-year loco-regional recurrence-free survival rate of 95% without high-grade late toxicity. Future prospective comparative evidence is important to ascertain the safety of dose de-escalation with regard to the current standard of 70 Gy.

### 3.2. Dose Escalation

Despite the significant improvement in loco-regional control with modern chemo–radiotherapy, dosimetric analyses have indicated that most local recurrences of NPC occurred within the high-dose CTV which received a full therapeutic dose of radiation [35]. This observation prompted interest in radiation dose escalation in selected tumors to improve loco-regional control further.

Several dose-escalation studies in NPC have reported encouraging treatment outcomes. These studies used standard anatomical imaging for target delineation and adopted a simultaneous integrated boost technique to treat the gross tumor. With regard to the GTV, these escalated regimens were typically hypofractionated at 2.17–2.42 Gy per fraction, prescribed at total nominal radiation doses of 66–76 Gy [35,36,37,38,39]. The reported short-term loco-regional control rates were high at 87–91%, yet no comparative studies were available to inform a clear benefit over standard non-escalated regimens. In addition, long-term toxicity data were unavailable due to inconsistent reporting and limited follow-up durations.

Instead of dose escalating the whole anatomically defined tumors, recent research has focused on the targeted image-guided dose painting of functionally active or radio-resistant areas. In a small randomized control trial which utilized ^18^F-PET as functional pre-treatment imaging, locally advanced NPC was treated by boosting areas with a standardized uptake value (SUV) ≥ 2.5 to 77Gy in 32 fractions [40]. The 3-year disease-free survival rate of 95.2% with this approach was superior to the rate of 79.2% obtained with conventional radiotherapy, providing preliminary evidence to support radiation dose escalation directed by functional imaging. Subsequently, in an observational study, ^18^F-PET was used to dose escalate radiotherapy for 101 loco-regionally advanced NPC [41]. By treating the GTV with 70.4–72.6 Gy in 33 fractions and boosting the sub-volume of gross tumors with ≥50% of the maximum SUV with 75.2–77.6 Gy, the 3-year disease-free survival rate was higher than that of the non-escalated control (87.9% vs. 82.4%), and there was no reported increase in high-grade adverse events.

Diffusion-weighted (DW) sequences from MRI have also been utilized to guide radiation dose escalation in NPC. A low apparent diffusion coefficient (ADC) value in DW-MRI is associated with adverse treatment outcomes independent of clinical stage and other known prognostic factors [42]. A recent randomized controlled trial of 260 patients reported improved disease-free survival with DW-MRI-guided dose-painting IMRT in loco-regionally advanced NPC compared with that of standard IMRT [43]. Following induction chemotherapy, patients in the experimental arm of this trial received 70.4–72.6 Gy in 32–33 fractions to GTV with an additional concomitant boost of 75.2–77.6 Gy to tumor areas with ADC below the mean value in the pre-induction images. This functional MRI-guided IMRT led to significant improvement in all survival endpoints with an excellent 2-year local recurrence-free survival rate of 100%. No significant increase in acute or late adverse events was reported.

It is noted that the comparable reported rates of radiation toxicities between dose-escalated and conventional IMRT should be viewed with caution, as the median follow-up durations of these studies were short at 2–3 years, within which radiation late effects are yet to manifest [44]. In addition, the lists of reported late complications in these studies were in many cases incomplete and non-exhaustive. Currently, the optimal SUV or ADC cut-offs to define boost volumes remain unclear, and the spatial and temporal variations of these metrics within tumors are also poorly characterized [45]. Further data, in particular 5- to 10-year survival rates, are required to inform practice as well as long-term safety with these dose-escalation approaches.

## 4. Sparing Organs at Risk

The introduction of IMRT as the standard radiotherapy technique for NPC has not only improved disease control, but has also reduced inadvertent radiation to multiple critical organs. The typical OARs of interest include the brainstem, spinal cord, optic structures, temporal lobes, auditory apparatus, pharyngeal constrictors, esophagus, larynx, oral cavity, salivary glands, pituitary gland, thyroid, and mandible [3,46]. Evidence from randomized controlled trials has demonstrated lower rates of physician-rated xerostomia and a superior preservation of salivary flow with IMRT than with 2D radiotherapy [47,48]. Incidences of multiple other late toxicities, such as temporal lobe necrosis, cranial nerve palsy, trismus, and neck fibrosis, were also reduced [49]. With the increasing awareness of late radiation complications and advances in radiotherapy dose optimization, researchers have strived to further reduce incidental radiation dose to specific OARs in NPC.

Post-radiation hearing deficit significantly impairs the quality of life of NPC survivors. The risk of hearing impairment after chemo–radiotherapy varies as a function of the pre-treatment status of auditory apparatus, radiation dose to the cochlea, and cumulative cisplatin dose. Given the current paradigm of intensive chemotherapy for localized NPC, the maximal prescribed cisplatin dose of 480–540 mg/m^2^ for patients with advanced diseases commonly reaches ototoxic levels [13,14]. It has therefore become prudent to enforce the superior protection of the auditory apparatus during radiotherapy planning. The QUANTEC and international guidelines have recommended that the mean cochlea dose be desirably kept below 45 Gy [3,50]. However, this threshold should not be viewed as risk-free, as the hearing impairment rate is still up to 30% for plans in which this criterion is fulfilled [51]. To achieve better cochlea-sparing, special optimization procedures with volumetric arc therapy may be considered [52,53,54]. By combining jaw closure, jaw tracking, a modification of the beam angle and a reordering of structure priority, mean cochlea doses could be significantly reduced as opposed to conventional planning without compromising PTV coverage, even in situations of advanced tumors with gross invasion to the petrous bone. These planning approaches, when used in combination, have been shown to reduce the mean cochlea dose by approximately 4–6 Gy. As the cochlea dose in NPC radiotherapy plans commonly falls at 40–50 Gy, where the slope of the normal tissue complication probability (NTCP) curve is steepest, even a slight decrease in the cochlea dose could result in a clinically meaningful reduction in the incidence of post-radiation hearing impairment. Furthermore, adopting volumetric arc therapy may also improve the dosimetric sparing of other OARs, such as the parotid gland, brainstem, and spinal cord, compared with step-and-shoot IMRT [55,56].

Radiation-induced cranial neuropathy in NPC survivors is another commonly overlooked late complication which can lead to a permanent detriment in speech and swallowing dysfunction in NPC survivors. In contemporary reports, the rate of long-term cranial nerve palsy in NPC survivors is as high as 5% with the hypoglossal nerve being one of the most commonly injured structures [1,57,58]. Traditionally, apart from the optic nerves, other cranial nerves are considered radio-resistant, and no special attempts have been made to protect them during radiotherapy planning. In a recent retrospective study, the maximal dose received by 1 cc volume (D1cc) of the hypoglossal nerve was found to be predictive of the risk of post-radiation hypoglossal nerve palsy in NPC survivors [59]. Hypoglossal nerve D1cc ≥ 74 Gy was associated with a high risk of future palsy (8-year; D1cc ≥ 74 Gy, 20.8%; D1cc < 74 Gy, 2.4%). By applying a dedicated dose constraint to remove hotspots within the hypoglossal nerves, neither the tumor target coverage nor dosimetric safety of other standard critical organs was compromised [60]. Its application should be routinely considered for NPC radiotherapy planning especially when dose escalation to the primary nasopharyngeal tumor is contemplated.

## 5. Future Directions

### 5.1. Adaptive Radiotherapy

Definitive radiotherapy for NPC is often associated with profound weight loss resulting from treatment-related mucositis, dysgeusia, nausea, anorexia, and saliva thickening. Acute changes in body contour could introduce inter-fractional inaccuracies in radiation delivery. These dosimetric uncertainties are particularly concerning due to the sharp dose gradients in IMRT, as a small topographic shift could lead to the geographic miss of targets or OAR overdose. Studies have demonstrated significant dose–volume changes in the brainstem, spinal cord, and parotid glands where radiotherapy plans were delivered without replanning in NPC patients who had significant acute weight loss [61,62].

Proactive adaptive radiotherapy by means of scheduled re-planning at mid-treatment may safeguard tumor control and reduce treatment toxicities. Compared with conventional treatment, the adaptive re-planning of definitive radiotherapy for NPC resulted in a higher quality of life and short-term loco-regional control (97.2% vs. 92.4%) in retrospective studies [63,64]. Target coverage and OAR dosimetry were also significantly improved [65]. Nevertheless, thus far, no prospective comparative trials on adaptive radiotherapy for NPC have been conducted. Although studies have shown that changes in body contour were most significant in patients with advanced tumors, a high pre-treatment body weight, and those who underwent concurrent chemotherapy [61], the optimal timing and patient selection for adaptive re-planning still remain unclear. Currently, adaptive radiotherapy is not yet to be routinely employed for all NPC patients, given its time- and resource-intensive nature. However, a scheduled re-plan may be considered between 15 and 25 fractions in high-risk patients [66]. Future technical advances in precise deformable registration and auto-segmentation may increase re-planning efficiency, facilitating its clinical implementation.

### 5.2. Particle Therapy

Photon-based IMRT is the current international standard radiotherapy delivery technique for localized NPC. Proton and heavy particle therapies offer the potential to further improve the therapeutic ratio due to their unique physical properties, such as a more focused deposition of the dose to the target volume with much-attenuated exit beams, thus minimizing the dose to adjacent OAR, and higher relative biological effectiveness compared with photon therapy. These intrinsic properties enable comparable (if not better) target coverage compared to that of photon-based techniques while reducing unintentional dose to OARs.

Clinically, several retrospective single-institutional studies have reported satisfactory treatment outcomes with proton therapy in NPC [12,67,68,69,70,71,72,73,74]. Although the delivery techniques (mixed photon–proton beams, double scatter technique, or intensity-modulated proton therapy (IMPT)) varied, the short-term loco-regional control rates consistently exceeded 80–90% in most retrospective series [75]. In the largest analysis to date, the outcomes of 80 patients treated with IMPT and 80 patients treated with photon-based IMRT were compared after propensity score matching [69]. The 2-year progression-free survival rates between the two groups were comparable. Patients treated with IMPT had fewer feeding tube placements and experienced less body weight loss than did photon-based IMRT patients, but the rate of high-grade radiation dermatitis was higher. Mixed photon and carbon-ion beam radiotherapy have also been investigated as primary treatments for localized NPC [76,77]. In one report from China, 69 patients with loco-regionally advanced NPC were treated with 56 Gy by photon-based IMRT followed by a carbon-ion boost of 15–17.5 GyE in 5–6 fractions [77]. This strategy led to a satisfactory 3-year progression-free survival rate of 85.2% with only two cases of grade 3 dermatitis.

In light of the dosimetric benefits and promising treatment outcomes of particle beam therapy for NPC, the National Comprehensive Cancer Network guidelines suggested the consideration of proton therapy where photon-based IMRT fails to fulfill normal tissue dosimetric constraints [78]. Nevertheless, the existing evidence to support the use of particle therapy in NPC remains derived from non-comparative studies of small sample sizes. It is unclear whether the dosimetric and radiobiological advantages would translate to superior long-term tumor control or a more favorable late toxicity profile. The current literature on particle therapy for NPC is further limited by short follow-up durations, the inconsistent reporting of adverse events, and potential temporal bias when historical photon-based IMRT cohorts were used as reference comparators. Well-designed prospective studies with detailed outcome reporting are crucial to formally determine and quantify the magnitude of benefit. Cost-effectiveness analyses are also important given the high costs incurred with these radiation delivery techniques.

### 5.3. Artificial Intelligence

Radiotherapy planning for NPC is considered a highly labor-intensive process which presently requires meticulous and time-consuming manual target delineation. The inter-observer variability of the contours of both tumors and OARs is also known to be high despite the availability of standard atlases [79]. Artificial intelligence (AI) has been increasingly investigated to enhance the efficiency of the planning workflow and to ensure the reproducibility of target and OAR contours [80].

Numerous studies have examined the role of AI-assisted target delineation for NPC [81]. The most widely studied area of application is the auto-segmentation of head and neck OARs. Given the relatively agreed-upon definitions of OAR boundaries in NPC radiotherapy planning, atlas-based auto-segmentation algorithms using deformable image registration have been developed [82,83,84]. These AI-generated OARs have excellent concordance with reference contours, and their reliability and reproducibility have been validated in large external cohorts. AI-based head and neck OAR auto-segmentation platforms are now commercially available and have greatly improved the efficiency of the radiotherapy planning workflow.

On the contrary, the application of AI in tumor target delineation for NPC is more challenging. Compared with normal structures, gross nasopharyngeal tumors and lymph nodes exhibit profound individual variability in shape, size, and anatomic extent. The highly variable nodal locations and indistinct soft tissue contrast in planning CT images were once obstacles to the development of the auto-segmentation of tumor targets in radiotherapy plans for NPC [85]. With technical advances in AI-based analytics in MRI, early success has been attained in the auto-segmentation of GTV for subsequent manual editing [86,87]. A fully automated deep learning-based model has also been employed to generate elective nodal CTVs for head and neck cancers [88]. These AI-generated nodal CTVs were highly reliable, requiring no or only minor manual edits in most of the contours. As of today, many of the current target volume auto-segmentation models in NPC remain validated only by limited single-institution data. Quality assurance and the exact workflow of clinical implementation require careful evaluation.

## 6. Conclusions

Radiotherapy for localized NPC is a complex treatment, the planning process of which requires careful execution to optimize tumor control and minimize radiation toxicities. Major advances have been made to refine target delineation, radiation dose modification, and plan optimization and delivery techniques (Table 1). Most of these studies were conducted in regions where EBV-associated NPC is endemic, and caution should be exercised when extrapolating these results in non-endemic areas. Future research should focus on the development of individualized treatment strategies in consideration of variations in patient factors, tumor extent, and inherent radio-sensitivity. Continual efforts to enhance the reproducibility and efficiency of the radiotherapy workflow are also crucial.

## Figures and Tables

**Table 1:** Summary of current radiotherapy considerations for nasopharyngeal carcinoma (NPC).

Components of Radiotherapy Planning for NPC	Areas of Consideration and Recent Developments
Target delineation (gross tumor volume (GTV))	- Reference to pre-treatment findings of high-resolution fiberoptic endoscopy, magnetic resonance imaging (MRI), and positron emission topography (PET) images - Input from diagnostic radiologists specializing in head and neck cancers
Target delineation (clinical target volume (CTV): primary tumor)	- CTV margin reduction - Sparing contralateral structures in unilateral diseases (i.e., gross tumor that does not cross mid-sagittal plane) - Reduction in CTV upon response to induction chemotherapy
Target delineation (CTV: regional lymphatics)	- Refinement of the delineation boundaries of nodal levels according to knowledge of nodal topographic characteristics - Omission of lower neck irradiation in uninvolved necks
Dose de-escalation	- Dose reduction from 70 Gray (Gy) to 60 Gy upon response to induction chemotherapy - The “gradient-dose” concept of delivering a dose gradient proportional to tumor volume and/or metabolic activity
Dose escalation	- Functional image-guided dose painting to sub-volumes of the target with a high tumor load or a radio-resistant region
Sparing of organ at risk (OAR)	- Cochlea-sparing intensity-modulated radiotherapy (IMRT) - Hotspot control of the hypoglossal nerve
Others	- Proactive adaptive radiotherapy - Clinical and radiation dosimetric benefits of particle therapies - Incorporation of artificial intelligence in auto-segmentation and plan optimization

## Data Availability

Not applicable.
